# Nestedness across biological scales

**DOI:** 10.1371/journal.pone.0171691

**Published:** 2017-02-06

**Authors:** Mauricio Cantor, Mathias M. Pires, Flavia M. D. Marquitti, Rafael L. G. Raimundo, Esther Sebastián-González, Patricia P. Coltri, S. Ivan Perez, Diego R. Barneche, Débora Y. C. Brandt, Kelly Nunes, Fábio G. Daura-Jorge, Sergio R. Floeter, Paulo R. Guimarães

**Affiliations:** 1 Department of Biology, Dalhousie University, Halifax, Nova Scotia, Canada; 2 Departamento de Ecologia, Universidade de São Paulo, São Paulo, São Paulo, Brazil; 3 Departamento de Física da Matéria Condensada, Universidade Estadual de Campinas, Campinas, São Paulo, Brazil; 4 Biology Department, Tropical Conservation Biology and Environmental Science, University of Hawai’i at Hilo, Hilo, Hawaii, United States of America; 5 Departamento de Biologia Celular e do Desenvolvimento, Universidade de São Paulo, São Paulo, São Paulo, Brazil; 6 División de Antropologia, Facultad de Ciencias Naturales y Museo, Universidad Nacional de La Plata, La Plata, Buenos Aires, Argentina; 7 Consejo Nacional de Inverstigaciones Cientificas y Tecnicas, Buenos Aires, Argentina; 8 Centre for Geometric Biology, School of Biological Sciences, Monash University, Clayton, Victoria, Australia; 9 Departamento de Genética e Biologia Evolutiva, Universidade de São Paulo, São Paulo, Brazil; 10 Departamento de Ecologia e Zoologia, Universidade Federal de Santa Catarina, Florianópolis, Santa Catarina, Brazil; Universidad Rey Juan Carlos, SPAIN

## Abstract

Biological networks pervade nature. They describe systems throughout all levels of biological organization, from molecules regulating metabolism to species interactions that shape ecosystem dynamics. The network thinking revealed recurrent organizational patterns in complex biological systems, such as the formation of semi-independent groups of connected elements (modularity) and non-random distributions of interactions among elements. Other structural patterns, such as nestedness, have been primarily assessed in ecological networks formed by two non-overlapping sets of elements; information on its occurrence on other levels of organization is lacking. Nestedness occurs when interactions of less connected elements form proper subsets of the interactions of more connected elements. Only recently these properties began to be appreciated in one-mode networks (where all elements can interact) which describe a much wider variety of biological phenomena. Here, we compute nestedness in a diverse collection of one-mode networked systems from six different levels of biological organization depicting gene and protein interactions, complex phenotypes, animal societies, metapopulations, food webs and vertebrate metacommunities. Our findings suggest that nestedness emerge independently of interaction type or biological scale and reveal that disparate systems can share nested organization features characterized by inclusive subsets of interacting elements with decreasing connectedness. We primarily explore the implications of a nested structure for each of these studied systems, then theorize on how nested networks are assembled. We hypothesize that nestedness emerges across scales due to processes that, although system-dependent, may share a general compromise between two features: specificity (the number of interactions the elements of the system can have) and affinity (how these elements can be connected to each other). Our findings suggesting occurrence of nestedness throughout biological scales can stimulate the debate on how pervasive nestedness may be in nature, while the theoretical emergent principles can aid further research on commonalities of biological networks.

## Introduction

Networks are pervasive in nature. Biological systems encompass multiple interacting elements that form networks across all organization levels, from molecules regulating metabolism of individual organisms to species interactions structuring ecosystems [1,2]. Biological networks share some common structural patterns, most notably: heterogeneity in the number of interactions per element [3] and formation of semi-independent groups of elements, called modules [4]. In contrast, other structural patterns may be characteristic of at least particular types of networks. This seems to be the case of nestedness, which occurs when interactions of less connected elements form proper subsets of the interactions of more connected elements [5,6].

The study of nestedness remains largely limited to ecological systems, particularly to systems formed by two non-overlapping sets of elements where interactions occur only between different sets, forming two-mode networks (e.g. [7]). For example, nestedness is routinely described in biogeographical systems in which sets of species occur in a set of habitat patches or islands (e.g. [8]), and in animal-plant mutualistic systems in which animal species pollinate or disperse the seeds of plant species (e.g. [7,9]). As originally used in biogeography, nestedness unraveled the structure of metacommunities in which species-poor sites presented subsets of the biota occurring in species-rich sites [8]. In mutualistic (as well as in parasitic and trophic) networks, nestedness revealed that the interactions of specialist species (the less connected) tend to be proper subsets of the interactions of generalist ones (the highly connected) [7,9–13]. These findings gave rise to hypotheses on the assembly mechanisms organizing ecological communities.

Nestedness also has implications for the ecological and evolutionary dynamics of these biological systems. In biogeography, a nested structure may indicate the source of colonizations, and/or the sequences of extinctions in metacommunities formed by habitat fragments [8]. Regarding species interactions, nested networks are generally robust against random extinctions of species [14,15], facilitate unstable dynamics in networks of antagonistic interactions [16], favor coevolutionary cascades in mutualisms [17], and influence the persistence of populations and the variation of individual fitness [18].

**Fig 1:**
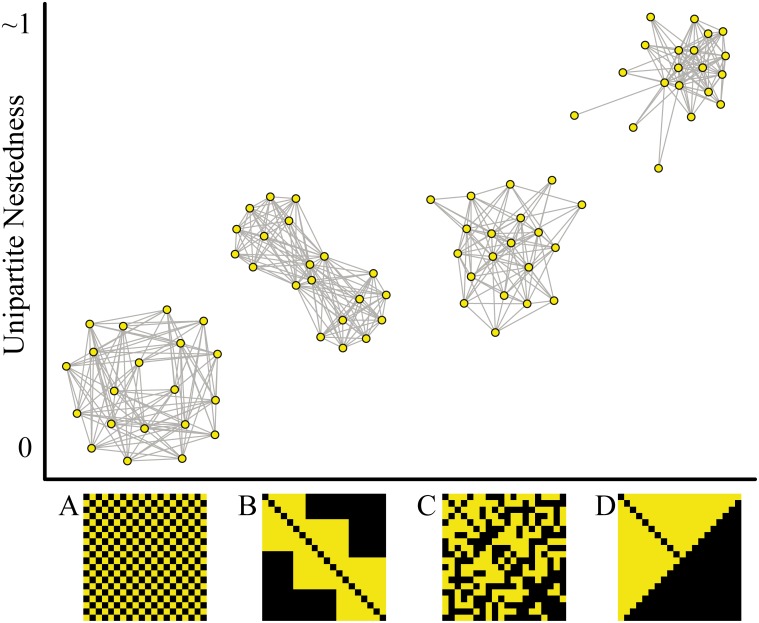
Nestedness of hypothetical undirected one-mode networks. Nestedness is zero when links are evenly distributed among nodes (A, checkerboard pattern), intermediate when links are clustered in distinct subsets of nodes (B, modular) or randomly distributed (C, random), and maximum when nodes with fewer interactions (peripheral nodes) interact with proper subsets of the subset of thighly connected nodes (D, perfectly nested). Networks have the same size (number of nodes) and connectance (proportion of realized links) and are followed by their binary adjacency matrices (symmetric and squared matrices, with yellow cells denoting link presence between nodes).

The discovery of nestedness as a ubiquitous pattern in two-mode ecological networks led ecologists to rethink the structure and dynamics of ecological assemblages. In nature, however, interactions are not always limited to occur between two well-defined sets of elements. Much more commonly, instead, biological systems form one-mode networks where all elements can in principle interact with each other. Moreover, the basic attributes of nestedness are not exclusive of two-mode networks. A nested one-mode network could display a gradient in the number of links, asymmetrical overlap among pairs of nodes, and a subset of highly connected nodes surrounded by nodes that interact to each other less frequently (Fig 1). However, only very recently these attributes began to be appreciated in one-mode networks, primarily in those describing trophic interactions at the species level [19,20] (but see [21,22]). One-mode networks encompass a much wider variety of biological phenomena occurring across all levels of biological organization—from intracellular molecular interactions all the way to ecosystems—leaving a key question unanswered: is nestedness a pattern present across biological scales?

Here, we investigate whether nested patterns are detected in biological networks across six levels of biological organization—molecular, individual, population, metapopulation, community and metacommunity. Our data illustrate biological systems as diverse as gene and protein networks, statistical networks describing phenotypes, animal social networks, gene flow among natural populations, food webs, and similarity of vertebrate communities at the biogeographical scale. From this diverse data collection, we show that nestedness spans over multiple levels of biological organization, and advance on theoretical common principles for the emergence of nested structures. Our focus, however, is on exploring what nestedness reveals about the organization and function of each of these disparate systems, with the ultimate goal of stimulating the debate on how pervasive nestedness may be in nature (e.g. [9,20,21,23–25]).

## Results

**Fig 2:**
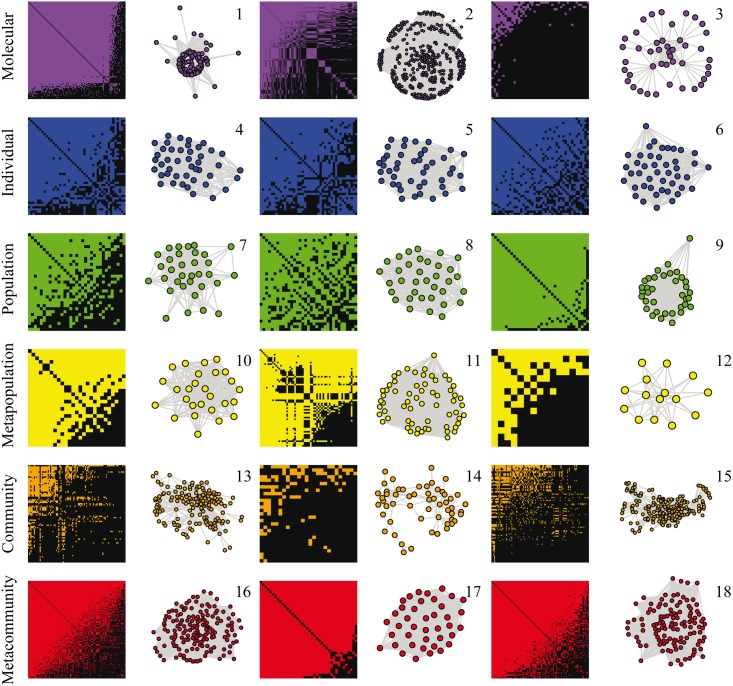
Adjacency matrices and network representation of 18 empirical biological systems across six organization levels. Molecular level: (1) yeast spliceosome protein-protein network, (2) *C*. *elegans* genetic network, (3) yeast nuclear exosome protein-protein network. Individual level: network representation of human crania in (4) men, (5) women and (6) men in Europe. Population level: social networks of (7) Guiana dolphins (*Sotalia guianensis*), (8) bottlenose dolphins (*Tursiops truncatus*) and (9) spotted hyenas (*Crocuta crocuta*). Meta-population level: genetic differentiation among populations of (10) frogs (*Pelophylax nigromaculatus*), (11), humans, and (12) house sparrows (*Passer domesticus*). Community level: food webs in coastal habitats, (13) Mangrove estuary, (14) Narragansett Bay, and (15) Florida Bay. Meta-community level: co-occurrence of reef fish species in sites, grouped by (16) taxon (genera), and two functional group schemes, (17) length and diet and (18) length, diet, mobility and school size. Matrices represent binary versions of the original weighted networks, in which colored cells denote presence of link between elements of the network, and black cells denote link absence. We used a successive threshold analysis (see Methods) to create binary versions of the weighted networks (for a similar approach see [26]); in this figure, we considered the version that maximized nestedness. Matrices were ordered by row and column totals for improve visualization of nested patterns, but the order does not affect the calculation of nestedness (see Methods).

We characterized the nested pattern in one-mode networks with a modified version of a widely used nestedness metric based on overlap and decreasing fill designed for two-mode networks [5,19] (see Methods). We detected significant nestedness in a diverse array of one-mode networks distributed across six different biological levels of organization (Fig 2).

**Fig 3:**
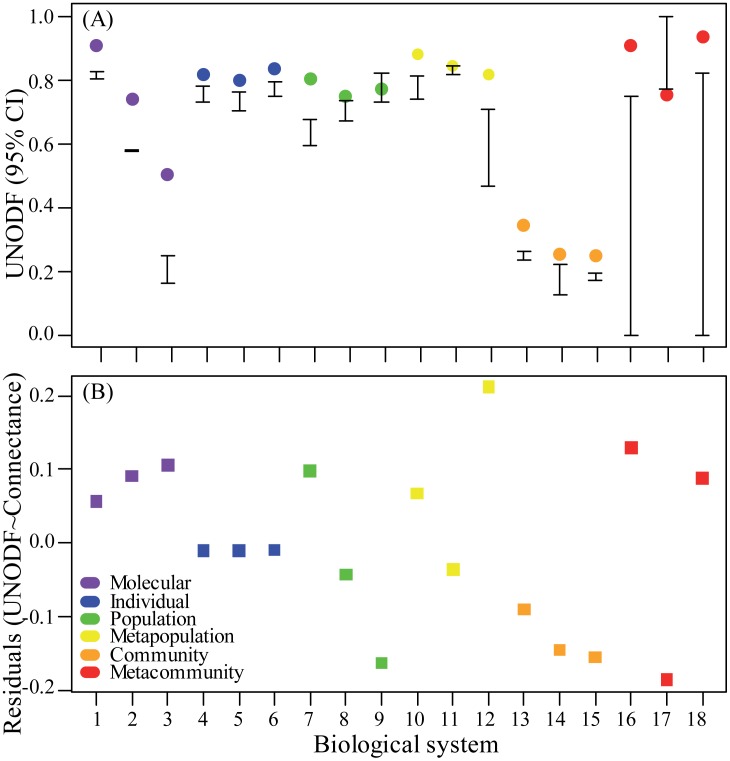
Nestedness (*UNODF*) of three representative one-mode biological networks for each of six organization levels. (A) Empirical *UNODF* (colored circles) and 95% confidence intervals generated by theoretical models (whiskers), with significant values falling outside the null expectancy. We binarized quantitative matrices with link weight cut-offs in ϵ[0,0.9] (see Methods), and the cut-off that maximized *UNODF* is presented here. For UNODF in all cut-offs, see Fig 4. *UNODF* among rows is equal to nestedness among columns (*UNODF*_*c*_ = *UNODF*_*r*_) for all undirected networks (but not for the directed networks 13, 14 and 15; see Fig 4) and only *UNODF*_*c*_ is shown. (B) *UNODF* corrected by network connectance. Since *UNODF* increases with network connectance (proportion of realized links in relation to possible links) (see S4 Fig), corrected *UNODF* is given by the residuals of the linear model of *UNODF* among columns and connectance (see S5 Fig). Networks are ordered according to biological scale, but the order within each scale is arbitrary. Number labels for the biological systems match Table 1, S1 Table, Fig 2.

The majority of these empirical one-mode networks (83%, n = 18) showed a maximum nestedness degree significantly higher than the null expectation (Table 1, Fig 3A), estimated by a theoretical model that considered the empirical number of nodes, connectance and the variance of the degree distribution (see Methods; [9]). We found no obvious trend of increasing or decreasing nestedness across organization levels (Fig 3A; Spearman correlation ρ = 0.031, S = 938.54, *p* = 0.901), even when accounting for variation in network connectance (Fig 3B; ρ = -0.254, S = 1215.1, *p* = 0.3092). This lack of relationship between organization level and nestedness degrees suggested that disparate biological systems from different scales may display similar nested structures.

**Table 1:** Nestedness (*UNODF*) of 18 biological one-mode networks across 6 organization levels.

	Organization level	Biological network	*UNODF*	*p*-value	Cut-off
1	**Molecular**	Yeast spliceosome proteins	0.91	< 0.001	0.0
2		*Caenorhabdtis elegans* genes	0.74	< 0.001	0.0
3		Yeast nuclear exosome proteins	0.50	< 0.001	0.0
4	**Individual**	Male human cranium	0.82	< 0.001	0.0
5		Female human cranium	0.80	< 0.001	0.0
6		European human cranium	0.83	< 0.001	0.0
7	**Population**	Guiana dolphin society	0.80	< 0.001	0.0
8		Bottlenose dolphin society	0.75	0.010	0.0
9		Spotted hyena social clan	0.77	0.700	0.0
10	**Metapopulation**	Insular frog populations	0.88	< 0.001	0.1
11		Global human populations	0.85	0.06	0.1
12		Insular sparrow populations	0.82	< 0.001	0.1
13	**Community**	Mangrove estuary food web, Consumers	0.35	< 0.001	0.0
		Mangrove estuary food web, Resources	0.47	< 0.001	0.0
14		Narragansett Bay estuary food web, Consumers	0.25	0.02	0.0
		Narragansett Bay estuary food web, Resources	0.21	< 0.001	0.0
15		Florida Bay food web, Consumers	0.25	< 0.001	0.0
		Florida Bay food web, Resources	0.25	< 0.001	0.0
16	**Metacommunity**	Reef fish functional groups (LD)	0.91	< 0.001	0.0
17		Reef fish functional groups (LDSM)	0.75	1.00	0.0
18		Reef fish taxonomic groups	0.94	< 0.001	0.0

**Fig 4:**
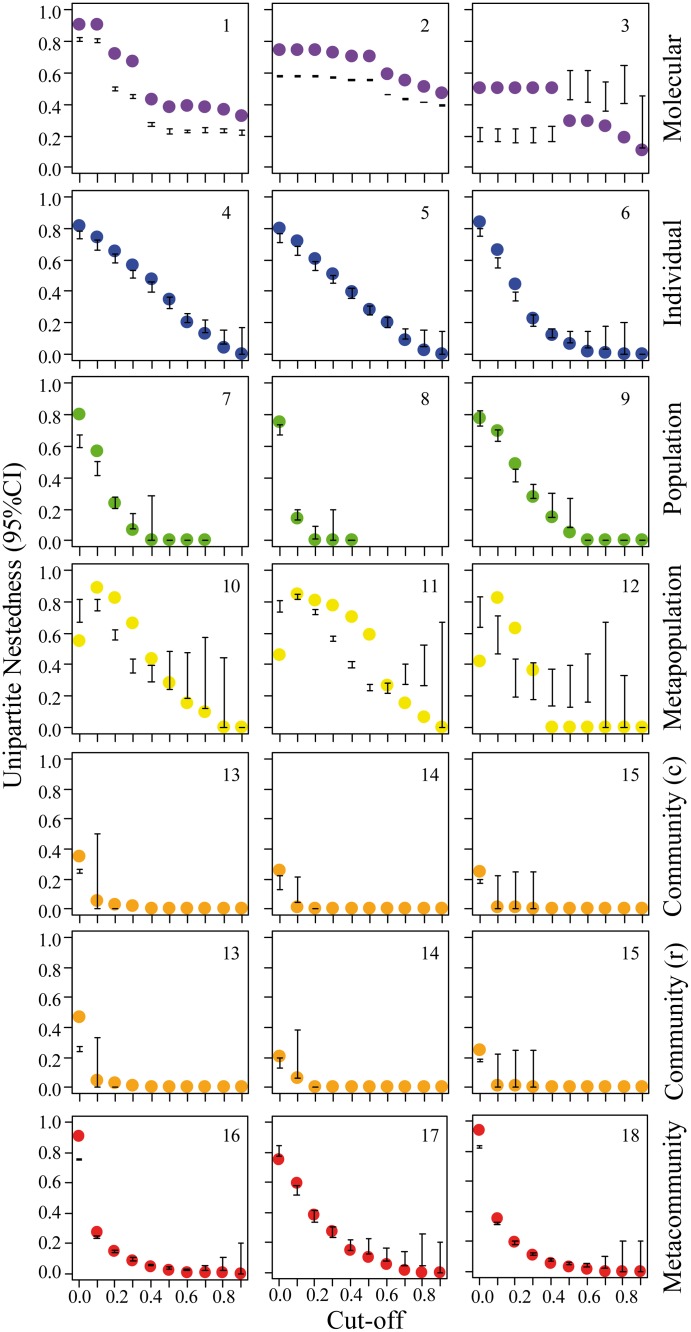
Nestedness (*UNODF*) across different cut-off levels for biological one-mode networks across six organization levels. We converted the weights of links in the original network to 0’s or 1’s using a successive threshold analysis (see Methods, S2 Fig). The *UNODF* values calculated for each cut-off (filled circles: empirical value) was compared with a benchmark null distribution generated by a null model (whiskers: 95% confidence intervals) that accounts for network size and connectance (see Methods). For the food webs (13–15), nestedness among rows (*UNODF*_*r*_) are different than nestedness among columns (*UNODF*_*c*_), whereas for the remaining undirected networks *UNODF*_*r*_ *= UNODF*_*c*_ (only one is displayed). Missing data represent networks completely dismantled at a given cut-off level.

*P*-values are based on null distributions generated by a theoretical model. Cut-offs denote the link weight used here to define a binary interaction in the network that maximized *UNODF* (for UNODF in all cut-offs, see Fig 4). For all undirected networks, *UNODF* across rows is the same as across columns and only one value is presented. For the directed networks (food webs 13–15), *UNODF* across rows denotes resources and *UNODF* across columns denote consumers.

We further explored the relationship between nestedness and other structural metrics known to recurrently occur in biological networks. The degree of nestedness was positively correlated with connectance (i.e. proportion of realized compared to possible links; S1A Fig). On the other hand, nestedness was independent of network size (number of nodes, S1B and S2 Figs) and not related to other commonly reported network patterns, such as network centralization and small-world properties (S3 and S4 Figs). Collectively, these results support the notion that nestedness is a distinctive aspect of the structure of one-mode biological networks that is not captured by other network metrics.

Since information on the frequency or strength of interactions can be key to understanding how biological systems function, we tested the sensitivity of *UNODF* to weighted data. We evaluated nestedness for binary networks generated after applying successive cutoffs in the original weighted networks (see Methods; for a similar approach see [26]). Generally, *UNODF* values peaked at low and decreased with high cutoffs for interaction strengths (Fig 4, Table 1). Regardless of the biological system and level of organization, nestedness tended to become non-significant with cut-offs higher than 0.3 (Fig 4). When no filter was applied (i.e., all interactions recorded are considered, cutoff = 0) some of the denser networks (e.g. the metapopulation level) displayed lower nestedness presumably due to weak links connecting peripheral nodes (Fig 4). On the other hand, with high cut-off values, the drastic removal of links from the network filtered off peripheral nodes and left only the core of strong connections, fading out the nestedness signal (Fig 4). This was particularly clear for sparser networks, such as food webs (S1 Table). These findings suggested that the nested architecture of these disparate biological systems depended on all links but especially the weak links that glue peripheral elements to the network.

## Discussion

We found nested architectures in one-mode networks depicting a wide range of biological systems. Nestedness spanning several levels of biological organization—from sub-cellular molecular interactions to the co-occurrence of vertebrates in biogeographical metacommunities—reveal shared features of the network organization of these biological systems: inclusive subsets of interacting elements with decreasing connectedness, asymmetrical node overlap, and a core-periphery organization. These findings contribute to the debate on the mechanisms that generate nestedness in natural systems [9,20,21,23–25].

### The emergence of nestedness

Nestedness implies a hierarchy in the linking rules of the networked system so there is heterogeneity in the number of interactions among its elements. The biological processes giving rise to nestedness can be multiple and non-exclusive, and certainly specific of each system [24,27–30]. In theory, these processes may impose constraints to the number of interactions an element of the system can have (leading to specificity) and to how these elements interact with each other (leading to affinity). Nestedness implies a gradient of specificity, and also particular affinity patterns, such that the highly specific elements tend to interact with (i.e. have greater affinity for) elements of low specificity. Therefore, in a nested network the highly connected core of nodes represents low-specificity elements, whereas the nodes in the periphery of the network represent high-specificity elements that interact with the highly connected core.

We can illustrate this theoretical scenario with well-studied two-mode networks depicting animal-plant mutualisms. Pollination by animals is a case in point. Successful pollination requires that species of plant visitors and flowering plants occur at the same place and make physical contact. While the probability of interaction is directly proportional to the local abundances of plant and pollinator species [29], flower morphology can impose species-specific constraints on number of animal species that can visit that flower. For instance, pollinators’ traits such as body length must be physically compatible with flowers’ traits, such as nectar holder depth and width [30]. Therefore, match in morphology (trait complementarity) and space (co-occurrence and abundance) define how species interact with each other (affinity) and so the number of interactions they can have (specificity). In a nested two-mode pollination network, this resultant interaction asymmetry includes a gradient of specificity in which the rare and/or more specialist species have more affinity for the more abundant and/or more generalist species.

Analogously, we hypothesize that the existence of constraints that determine specificity and affinity can be thought as an overarching feature of nested one-mode networks. Any one-mode biological system may contain elements with different specificities and affinities; but when there is a gradient of specificity among elements that have different affinities for each other in a way that highly specific elements have greater affinity for less specific elements, the system would be nested. Here, we investigated the emergence of nestedness in 18 one-mode biological systems across six different levels of biological organization (S1 Table). In the following section, we explore for each of these systems possible mechanisms generating constraints in specificity and defining affinity patterns that lead the emergence of nestedness. As these biological processes are system-dependent, often unknown and likely complex, some of our propositions are necessarily speculative at the moment. Pinpointing the definitive mechanisms for each system is beyond the scope of this study, but we hope these hypotheses help to point out research directions.

### Nestedness across biological organization levels

#### Molecular level

We analyzed networks of interacting proteins of the spliceosome and nuclear exosome of budding yeast *Saccharomyces cerevisiae*; and network of genes of the worm *Caenorhabditis elegans*. Nestedness in *S*. *cerevisiae* was first reported by [28] and associated with affinitity and specificity. In fact, differences in connectivity among spliceosome or exosome proteins leading to nested networks may be generated by physical constraints determining the specificity of the protein interaction such as size and available surface for interaction. This might also be modulated by functional features, for example the specific time when a protein is incorporated and catalytic activity is triggered [28]. Although core proteins are important for catalysis and structural properties of most macromolecular complexes, the nested structure implies that peripheral-core interactions are common. As a consequence of these asymmetrical interactions, dysfunctional peripheral or transient elements likely indirectly affect the whole network via their direct interactions with core proteins [28]. In yeast spliceosome, Cwc24p has only a few interactions in the spliceosome network, but it is necessary to stabilize the U2 snRNP association to the spliceosome and its absence abrogates splicing in transcripts with non-consensus branchpoint sequences [28,31]. Also, proteins transiently associated to the nuclear exosome, for example, can play important roles in the regulation of its catalytic activity. Nip7p is one of these factors, conserved throughout all eukaryotes and archaea, and in the archaea *Pyrococcus abyssi* interaction of Nip7p and Rrp43p has important functional implications for exosome activity [32]. Similarly, the genetic network of *C*. *elegans* [33] depicted mainly gene interactions based on signaling pathways, which in fact comprise the majority of interactions in the cell. The nested pattern suggests that the signaling pathways are organized in such a way that some genes showing more specific signaling pathways interact with a subset of more interactive genes.

#### Individual level

We analyzed morphological networks of individual human crania. Complex morphological structures form anatomical networks in which the statistical association between morphological traits is a result of developmental constraints, such as those imposed by spatial contiguity and developmental interactions during ontogeny [34]. Network representations of the human cranium showed a significant level of nestedness, likely due to the hierarchical characteristics of the cranium development [35]. Less connected cranial morphometric variables such as measurements describing more local morphological units (e.g. orbit, zygomatics) could be linked by a subsample of the links observed among the most connected cranial measurements at larger scales (i.e. the cranial measurement describing more global characteristics, such as the face). The pattern of associations at this larger scale is more influenced by factors determining the growth of the organism as a whole [35,36].

#### Population level

We analyzed social interactions among animals living in societies with fission-fusion dynamics (i.e. with groups that vary in membership and spatial cohesion over time; [37]). In these social networks, individual variation in sociality and/or residency can constrain number of social relationships. Variation in sociality affects social choices directly: some individuals are more gregarious or more prone to interact with “popular”, high-rank individuals (see [38]). Variation in residency affects social choices indirectly: when individuals differ in the time they spend in an area, there are unequal opportunities for social interactions [39]. Our study cases exemplify these two possibilities. The spotted hyena clan is a hierarchical society containing matrilineal kin groups, in which adult females vary in social dominance and priority of access to resources [40]. In contrast to other networks in which nestedness was (at least partially) a consequence of weak links, nestedness in the hyena society was detected only after some of the weaker social interactions were filtered off (i.e. significantly higher nestedness for cut-off = 0.3). We hypothesize that these weak interactions that swamped nestedness are short-term and among both kin and unrelated individuals that routinely form and dissolve, usually in response to prey abundance and competition [40]. In the hyena society, nestedness would indicate that strong social interactions among individuals are very asymmetrical, potentially due to hierarchy and matrilineal ties.

In contrast, dolphin societies are egalitarian and more dynamic, with much more ephemeral groupings and fluid social relationships [39,41]. As spatial proximity affects the structure of dynamic societies [39], individual variation in residence affects the number and strength of dolphin relationships. Both the bottlenose and Guiana dolphin populations analyzed here contain a set of resident individuals (who have many opportunities to interact among themselves) along with transient dolphins (who span over larger areas, and so interact with the social core sporadically when passing through the area and assume peripheral roles in the social network). The weaker links promoted by transient individuals contributed to nestedness of these dynamic social systems. Overall, nested societies should be heterogeneous, with potential implications to the flow of genes, information, and diseases [42].

#### Meta-population level

We analyzed networks of populations linked when sharing genetic variants. Here, the dispersal ability of the species constrains gene flow, restricting the number of links between populations. Population expansion may generate a gradient in the number of links. As the range of a species increases, populations go through successive subsamplings of genetic variability (founder effect), resulting in populations with little genetic variation possessing a subset of the variants present in more genetically diverse populations. Therefore, nestedness may indicate that dispersal of individuals is not sufficient to compensate the genetic diversity eroded by genetic drift or selection during the increase of species distribution. The genetic metapopulation networks of insular frogs and house sparrows we studied were nested. In both cases, the metapopulations were distributed in archipelagos spreading around 50-100km [43,44] and these species show limited dispersal capabilities. For example, house sparrows generally cover very short travel distances [45] and thus the limited dispersal may lead towards less genetic variability as islands are farther from the continent. Accordingly, insular frogs are intolerant to seawater, limiting dispersal and eliminating any source genetic diversity that could counteract the random genetic drift [44]. The lack of nestedness in genetic metapopulation networks can also provide information on how the complex interplay between spatial scales and dispersal ability shapes genetic diversity. The genetic metapopulation network of humans was not nested, even though human populations have undergone successive genetic bottlenecks after leaving Africa [46]. We hypothesize that the recent population expansion and high dispersal capability of humans increased the gene flow among geographically close populations. This decreased population differentiation to a degree that is high enough to obscure patterns of nestedness at the metapopulation level, which could still exist at broader scales (among continents; e.g. [47]). Moreover, gene flow within continents may contribute to reduce the signal of bottlenecks potentially generating compartments in the human metapopulation network.

#### Community level

We analyzed networks depicting food webs, in which a suite of species traits may constrain their trophic interactions. For instance, the niche breadth of a species can be defined by body size, gape size and ecophysiological tolerances [48]. A central problem in feeding ecology at the community level is to understand how species share resources, especially in the context of niche theory [49]. Nestedness provides a quantitative measure of overlap in relation to the interaction structure of multispecific assemblages, unraveling the architecture of niche overlap and so providing insights into the processes shaping niche organization in a multispecific context. In the three food webs herein analyzed, nestedness only appears when we consider weak interactions. Theory suggests nestedness can reduce the likelihood of stability in systems of antagonistic, trophic interactions [16]. Conversely weak links may promote stability in food webs [49,50]. By affecting how the channels of slow energy flow generated by weak links are distributed in food webs [50,51], nestedness may have relevant and unanticipated consequences for community stability.

#### Meta-community level

We analyzed networks representing taxonomic and functional diversity similarity of communities of reef fishes. We found nested communities across a gradient of both taxonomic and functional diversity along the western Atlantic. Our results corroborate the scenario of the richness gradient observed from the richer (both taxonomic and functional perspectives) center of diversity (Caribbean) to depauperate peripherical areas (e.g. the Brazilian coast or isolated oceanic islands in the western Atlantic [52,53]. The nested pattern in taxonomic and functional groups may be an indicative that biodiversity is primarily composed of few key clades that quickly saturate the ecological space. Innovations (the peripheral nodes) would represent secondary additions to the network structure, both in terms of genera (phylogenetic conservatism) and functional groups (niche conservatism) [54,55]. They may also result from environmental constraints (e.g. temperature fluctuations) controlling what can evolve in the spatial periphery of a metacommunity, and isolation gradients [53]. This scenario agrees with the majority of reef fish families being present in virtually all tropical regions of the globe [56] and with rare species representing vulnerable functions in reef fish communities [57].

### Caveats and the way forward

Combined, these study cases suggest that system-specific interaction constraints may make nestedness a recurrent in biological networks at multiple levels of organization when both strong and weak interactions are taken into account. While common in bipartite biological networks (e.g. [9,23,24]), the recent search for nestedness in unipartite systems suggest it may not be as widespread as earlier thought [20,21]. Here, we temper these recent findings by reopening the possibility for nestedness to be present in very disparate systems throughout biological scales. There are, however, three caveats that leave the definitive assessment of pervasiveness of nestedness in nature an open question.

First, in our study we used an adapted version of the most used nestedness metrics (*NODF*, [5]) for one-mode networks [19]. But there are different analytical methods to compute nestedness [20,21,25,27]) and despite recent reviews (e.g. [10]) there is no consensus in the literature on which metric best describes nestedness. Second, our dataset is extensive and diverse, yet not comprehensive. We aimed to represent very different natural systems with which we are familiarized, so to discuss what nestedness reveals about their organization and function, but an exhaustive analysis of nestedness within scales was beyond the scope of this study. This leads to the final caveat. Since specificity and affinity among elements are intrinsic features of each system, the proposed general mechanisms assembling interactions into a nested architecture remain tentative. A wider investigation is warranted to reveal whether nested architectures are counter-shaded in other biological one-mode networks. We invite researchers to investigate proximate and ultimate processes that could generate nestedness in their biological networked systems of interest. These, once illuminated, will cast more light on the contribution of specificity and affinity as overarching mechanisms generating nestedness.

## Conclusions

We showed nestedness in a diverse collection of networks characterizing natural systems at multiple biological scales, and discussed the implications of the nested structure for each case. We also hypothesized that nestedness emerges across scales due to processes that, although system-dependent, may share a general compromise between two features: specificity (the number of interactions the elements of the system can have) and affinity (how these elements can be connected to each other). Our findings advance the idea of nestedness throughout biological organization levels, which fuel the debate on how pervasive nestedness may be in nature. However, it is still early to pinpoint definitive processes generating nestedness in each biological system; as well as to layout the unifying rules assembling elements of biological systems into a nested architecture. Our hope is to provide starting points for case-specific, process-oriented and cross-disciplinary research to shed more light onto commonalities of life across multiple scales.

## Materials and methods

### Empirical data

We analyzed 18 biological systems representing six levels of biological organization (S1 Table). At the *(i) molecular level*, we investigated interactions among proteins and among genes. We explored two protein-protein networks in the budding yeast *Saccharomyces cerevisiae*: first, the network formed by proteins related to the macromolecular complex spliceosome [58] and, second, the exonucleases complex exosome. The spliceosome is a multi-megadalton machinery composed of RNAs and more than 100 proteins. Most of these proteins are conserved from yeasts to humans [59,60]. We retrieved 103 spliceosome proteins from the STRING database [61], to build a weighted one-mode network, in which individual proteins are nodes linked by the level of support for the occurrence of interaction between them (i.e. probability that interaction is correct). This reliability score comes from the combination of different experimental evidences [62], and goes from zero (no evidence) to one (strong support for interaction) (additional details in [28]). Likewise, we built a weighted one-mode network with 44 proteins from the yeast nuclear exosome, retrieved from the IMEx database [63,64]. A third subcellular network depicted interactions among the genes of the worm *C*. *elegans*, a very reliable whole-animal model from which the largest metazoan genetic interaction network has been decoded [33]. Using the systematic interaction analysis (GSI), 11 query genes mutations (most of which is involved in signaling pathways) were tested for genetic interactions with 454 target genes [33,65]. The one-mode genetic interaction network contained the target genes as nodes linked by the interactions they shared with query genes, quantified by the Simpson index (as recommended in [66]).

At the *(ii) individual level*, we investigated network representations of a complex morphological structure, the human cranium. In this morphological network, nodes represented anatomically defined measurements between landmarks points, and links represented the Pearson correlation coefficients among the measurements between the landmarks [67]. Therefore, the network represent how integrated is cranial morphology and development. We analyzed 44 cranial measurements (obtained by [68]) from a sample of 1367 male and 1823 female individuals from 30 recent modern human populations distributed worldwide [68]. We explored three cases: the interpopulation morphology for females, for males, and within-population morphology. In the first and second networks, we averaged the 44 cranial measurements for each of the 30 populations (two 44 by 30 matrices); in the third network, the 44 measurements were correlated among 164 individual males from Europe (44 x 164 matrix) (Norse: Medieval samples from Oslo; Zalavar: recent samples from Hungary; and Berg: recent samples from Carinthia, Austria).

At the *(iii) population level*, we investigated social interactions among individuals of three species that live in societies with fission-fusion dynamics: spotted hyenas *Crocuta crocuta* [40,69], Guiana dolphins *Sotalia guianensis* [39], and bottlenose dolphins *Tursiops truncatus* [41]. These animal societies were represented as social networks in which nodes depicting identified individuals were linked by their social relationships, the strength of which was inferred based on co-occurrence in groups. Individual hyenas were identified by their unique spot patterns [40]. Individual dolphins were identified by natural markings on the dorsal fin using standard photo-identification protocols [39,41]. Dyadic associations were estimated as the proportion of times individuals were observed together [38], using the twice-weight index (TWI) for hyenas [40] and half-weight index (HWI) for dolphins [39,41]. We avoided spurious associations among hyenas by analyzing only individuals observed more than five times, all from a single, well-sampled clan [40]. For dolphins, we analyzed only individuals seen using the study area during the study period more than three times [39,40].

At the *(iv) meta-population level*, we investigated gene flow among 62 human populations worldwide (43 populations with more than 10 unrelated individuals from [47,70]; 19 populations with more than 10 unrelated individuals from [71]), among house sparrow populations from 15 Norwegian islands [43,72] and among 27 pond-frogs populations in continental and insular China [44,73]. In these networks, nodes represent populations and are linked by their genetic similarity inferred through variance of microsatellites alleles size, measured by 1-R_ST_ [74]. The human dataset consists of 678 autosomal microsatellite loci; the house sparrow dataset contains 13 microsatellite loci, all nuclear and selectively neutral; and the frog dataset consisted of nine neutral microsatellite loci.

At the *(v) ecological community level*, we investigated food webs representing trophic interaction among species. Food webs are networks of species depicted as nodes, which are connected by trophic interactions [75]. Since species can be both consumers and resource at the same time, food webs are better described as directed networks where nodes may be connected via in- and out-links. Here we focus on 3 food webs of coastal species and energy flux between them, which were chosen for varying in size (small: 29, medium: 77, large: 109 nodes) (S1 Table) and for being widely used in the literature because of their good resolution and sampling.

Finally, representing the *(vi) meta-community level*, we investigated the taxonomic and functional diversity of reef fishes along the Western Atlantic, including communities in the North-western Atlantic, Caribbean and the South-western Atlantic (2, 5, and 17 communities respectively). Underwater visual surveys were conducted using strip transects (20x2m) on shallow reefs (depth<20m), where all individuals (n = 536,378) were counted (total length estimated in 10-cm bins) and identified to the species level [76]. Species were grouped taxonomically at the genus level (161 genera) and using two functional grouping (FG) schemes, i.e. combination of traits: Diet x Body Size Class (40 FGs) and Diet x Body Size Class x Mobility x School size (122 FGs) (see [77] for full description and relevance of the chosen functional traits in reef fishes). Genera or functional groups were depicted as nodes connected by co-occurrence in the sampled communities given by the quantitative asymmetric Bray-Curtis index of similarity. The index was corrected using size-corrected body mass [78] since it accounts for biological turnover among individuals of different mass and differences in size structure among different communities [79].

### Unipartite nestedness

Biological systems can be depicted as networks in which nodes represent elements of the system and the links between nodes describe interactions among these elements. A network can also be represented as an *m* × *m* adjacency matrix **A,** where *m* is the number of elements in the system. Each matrix cell *a*_*ij*_ is filled with 1 if the element represented in column *j* interacts (or is related to) with the element represented in row *i*, and is filled with 0 otherwise. The nature of interactions defines whether a network is directed or undirected (i.e., symmetric or asymmetric interactions), weighted or binary (i.e., quantitative or qualitative interactions), one- or two-mode (i.e., the network comprises a single set of interacting nodes, or two distinct sets) [80]. Nestedness had been traditionally assessed in binary two-mode networks (e.g. [8,9]), commonly via the Nestedness metric based on Overlap and Decreasing Fill (*NODF* [5]). Only recently, methods for assessing nestedness in direct and undirect one-mode networks have been introduced [19–21]. However, each metric reveals different properties of nestedness, for instance node overlap or segregation [20], and heterogeneous degree distribution or assortativity [21]. Given how widely used *NODF* is, we understand that an explicit generalization of this metric for one-mode networks is warranted. We present here the *UNODF*, the Unipartite version of the *NODF* metric.

*NODF* is based on two properties: paired overlap and decreasing fill [5]. The algorithm to compute *NODF* considers an incidence matrix with *m* rows and *n* columns (i.e. defining a two-mode network). The overlap in interactions of any two nodes, *w* and *z*, is computed depending on the relative position of the rows or columns and the number of interactions (i.e. degree) of each node, *k*_*w*_ and *k*_*z*_. Assuming *w* < *z* (i.e. *w* represents a row located at an upper position from row *z* or a column located at a left position from column *z*) and *k*_*w*_ > *k*_*z*_ the pair conforms to the notion of decreasing fill and *DF*_*wz*_ = 100; however, if *k*_*w*_ ≤ *k*_*z*_ then *DF*_*wz*_ = 0. The paired overlap between *w* and *z* (*PO*_*wz*_) is the proportion of interactions the row/column *z* shares with *w*. The degree of paired nestedness *N*_*wz*_ = 0 if *DF*_*wz*_ = 0 and *N*_*wz*_ = *PO*_*wz*_ if *DF*_*wz*_ = 100. NODF is then computed as the average of *N*_*wz*_ considering all pairs of rows and columns *w* and *z*.

Because *NODF* ultimately depends on pairwise comparisons there is no reason to restrict the metric to two-mode networks. Based on the algorithm proposed by Almeida Neto et al. [5], Lee et al. [19] adapted the *NODF* metric to be used in one-mode networks by defining:

$$S=\frac{2}{N\left(N-1\right)}\sum_{i}^{N}\sum_{j,\;i<j}^{N}\frac{\sum_{l=1}^{N}a_{il}a_{jl}}{\text{min}\left(k_{i},k_{j}\right)}$$ (1)

where *N* is the total number of nodes of the network; **A** is a *N* × *N* matrix with all elements (*a*_*ij*_) represented in the rows and columns; $k_{i}=\sum_{l=1}^{N}a_{il}$ is the degree of node *i*. We noticed that *S* considers overlap between all nodes, even when these have the same degree (*k*_*i*_ = *k*_*j*_). However, a given set may only be a proper subset in a larger set. Thus, we adapted the metric *S* so that the overlap is always unidirectional, computing the proportion of the interactions of the node with the smaller degree that are also present in the set of interactions of the node with larger degree (i.e. node pairs with same degree are not taken into account). Therefore, we redefine the metric *S* as the Unipartite Nestedness based on Overlap and Decreasing Fill (*UNODF*):

$$UNODF=\frac{2}{N\left(N-1\right)}\sum_{i}^{N}\sum_{j,\;i<j}^{N}\frac{\sum_{l\neq i,j}^{N}(1-\delta_{k_{i},k_{j}})a_{il}a_{jl}}{\text{min}\left(k_{i},k_{j}\right)}$$ (2)

where $\delta_{k_{i},k_{j}}$ is the Kronecker delta which means that $\delta_{k_{i},k_{j}}=1$ if *k*_*i*_ *= k*_*j*_, and $\delta_{k_{i},k_{j}}=0$ otherwise.

### Properties of unipartite nestedness

In completely non-nested one-mode networks *UNODF* = 0, while in perfectly nested networks *UNODF* tends towards 1 (Fig 1, S2 Fig). Because interactions of an element *i* with itself (*a*_*ii*_ = 0) are not considered, *UNODF* will only approach 1 for large matrices that are highly nested. Since *UNODF* is based on pairwise nestedness, each pair of nodes in a perfectly nested matrix is nested within each other (a staircase-like pattern); and the calculation of the nestedness value is independent of the ordering of the network adjacency matrix (different than the original *NODF* [5,25]).

In an adjacency matrix representing one-mode networks, all elements are represented as rows and also as columns. Thus, if such a matrix is symmetrical with respect to its main diagonal, which is the case for undirected networks, computing nestedness among rows or columns will result in the same value. Yet, the interactions depicted in matrix elements *a*_*ij*_ and *a*_*ji*_ represent different things in directed networks. In a food web, for instance, a presence in *a*_*ij*_ could represent the consumption of the species *j* by the species i, and *a*_*ji*_ depict the consumption of *i* by *j*. For this reason, directed networks will have two different *UNODF* values (and interpretations) if *UNODF* is obtained by computing the pairwise overlap among columns or among rows. For example, in a food web where species in rows consume the species in columns, nestedness among rows, *UNODF*_*r*_, measures nestedness in resource use overlap among consumers; conversely, nestedness among columns, *UNODF*_*c*_, describes the extent to which resources or prey share common consumers in a nested fashion. Additionally, food webs are the only case studied here in which a node (species) could interact with itself (cannibalism: *a*_*ii*_ = 1). However, because cannibalism was absent in our data sets, here we disregarded self-interactions *a*_*ii*_ = 1 for the sake of generality.

The choice of how to consider link weight in nestedness computation for weighted networks depends on network type and the biological meaning of the weights. As link weights are context-dependent and have very different meanings across systems (S1 Table), we focused on binary networks. We performed a sensitivity analysis to evaluate the effect of weighted links in the detection of nestedness. We evaluated the behaviour of *UNODF* under successive link weight thresholds (cut-off values) used to define a binary matrix (as in [26,28,67]). To allow for cross-system comparisons, we first standardized the link weight distribution of all weighted networks by dividing link weights by the maximum weight of each system, so that *a*_*ij*_ ϵ [0,1]. We subsequently computed *UNODF* for 10 binary networks for each system (S1 Table), one for each of the 10 weight cut-offs defined at 0.1 intervals (note that the cut-offs have no units). For example, for a cut-off of 0.3, all links with weight below this limit were filtered off (*a*_*ij*_ < 0.3 → *a*_*ij*_ = 0) or otherwise maintained (*a*_*ij*_ ≥ 0.3 → *a*_*ij*_ = 1) (S5 Fig). If nestedness is present regardless of value used as a threshold to build binary networks, the system can be regarded as nested despite link weight heterogeneity.

To evaluate if *UNODF* would capture distinctive topological features that add to other commonly analyzed properties of one-mode networks, we tested the correlation between the *UNODF* of all empirical networks with six network metrics (mean degree, betweeness centralization, closeness centralization, eigenvector centralization, mean clustering coefficient, mean shortest path length) (S3 Fig). To further explore this, we fitted a simple linear model to *UNODF* and the first principal component (from principal component analysis) summarizing the six centralization metrics of all 18 networks (S4 Fig).

### Significance tests

We assessed nestedness significance with a null model, comparing the degree of nestedness computed for each empirical network to a benchmark distribution of nestedness values calculated for theoretical networks. We used a theoretical model widely used for examining the significance of structural patterns in two-mode biological networks (null model 2 in [9]), in which the probability of a link connecting two nodes is proportional to the number of links observed for both nodes. This model restrains the empirical network size (number of nodes), connectance (proportion of realized links) and the heterogeneity in the degree distribution (without fixing the degree of each node). We adapted this probabilistic null model for one-mode networks, whose adjacency matrix is squared, symmetric and the diagonal is zeroed. For each empirical network, we created 1,000 theoretical networks for each of the binary adjacency matrices resultant from the 10 weight cut-offs.

A network was considered significantly nested whenever the value of the observed network lied outside of the 95% confidence intervals of the benchmark distribution. Since the null model relies on the empirical data to build the theoretical networks it attempts to account for passive sampling artefacts and disentangle nestedness from sampling biases. Finally, since the effect of connectance on nestedness is often undesirable [20], we accounted for the relationship between *UNODF* and connectance (S1A Fig) with this null model that replicates the connectance of empirical networks in the theoretical networks (see also [20]).

### Data and code availability

The nestedness metric and null model are available as the R package *UNODF 1*.*2* deposited at https://bitbucket.org/maucantor/unodf. All data sets and the code to reproduce the analyses and figures are available for download in the repository https://bitbucket.org/maucantor/unodf_analyses/src.

## Supporting information

S1 Fig Relationship between Unipartite Nestedness (*UNODF*), network connectance and size. (A) *UNODF* and network connectance (proportion of realized links in relation to possible links); and (B) *UNODF* and network size (number of nodes). A simple linear regression suggests that *UNODF* increases with connectance (*R*^2^ = 0.73, *p*<0.0001) but not with size (*R*^2^ = 0.07, *p* = 0.763). Colored points represent empirical nestedness value and whiskers show the 95% confidence interval of *UNODF* computed for theoretical networks generated using the null model. Values outside of the 95% Confidence Intervals are significant. Note that only nestedness among columns (*UNODF*_*c*_) is displayed, since for all networks (except food webs, Community level) *UNODF*_*r*_ *= UNODF*_*c*_.
(DOCX)

S2 Fig Relationship between Unipartite Nestedness (*UNODF*) and size of theoretical nested one-mode networks. (A) *UNODF* and size (number of nodes, *n*) of perfectly nested networks. (B) Adjacency binary matrices (yellow cell = 1, blue = 0) of representative small networks (20 > *n >* 3) and their respective unipartite nestedness values among columns and rows (*UNODF*_*r*_ *= UNODF*_*c*_). The *UNODF* metric is sensitive to very small networks, but becomes asymptotic for networks with more than 10 nodes. Note that *UNODF* tends towards—but does not reach—1 because we considered undirected one-mode networks without nodes that interact with themselves. Therefore, the diagonal of the network adjacency matrix A is zeroed (*a*_*ii*_ = 0). Few cases in which a node can have a link with itself include cannibalistic events in food webs. However, for the sake of generality across biological systems, these cases were disregarded here.
(DOCX)

S3 Fig Network metrics describing the centralization of the network and Unipartite Nestedness (*UNODF*) in one-mode networks. Centralization is measured here by 6 metrics (colored lines) for both theoretical (perfectly nested, Barabási, Erdös-Rènyi) and empirical networks (three examples for each of the six biological levels: molecular, individual, population, metapopulation, community, metacommunity; see S1 Table). In (A) the x-axis corresponds to the Unipartite Nestedness metric defined in the main text. In (B), x-axis contains the residuals of the linear regression between Unipartite Nestedness and network connectance (proportion of realized links in relation to possible links) given the positive relationship between them (see Fig 2, S4 Fig). We found no relationship between nestedness and centralization metrics, suggesting that *UNODF* captures a distinct topological pattern (see also, S4 Fig). Samples are connected simply to make it easier to read the trajectories of each metric.
(DOCX)

S4 Fig Relationship between Unipartite Nestedness (*UNODF*) and network centralization, as given by a simple linear model. Here, we corrected *UNODF* for network connectance using the residuals of the regression between *UNODF* and connectance; see S5 Fig. Centralization was described using the first principal component to summarize (A) all 6 centralization metrics, (B) the centrality metrics (degree, betweeness, closeness, eigenvector centrality) and (C) small-world properties (Clustering coefficient and shortest path length). In all cases, the metrics were not related to *UNODF* (All metrics: *R*^2^ = -0.04, *p* = 0.678; Centrality: *R*^2^ = 0.06, *p* = 0.146; Small world: *R*^2^ = 0.01, *p* = 0.312), suggesting that *UNODF* captured a topological feature of one-mode network different than centralized networks or with small world properties.
(DOCX)

S5 Fig Methodological approach to define a binary interaction in weighted networks. Color code corresponds to the strength of the interaction between elements. Hypothetical square adjacency matrices in which (A) the interactions between elements of a one-mode network are filtered off according to cut-offs (*x*) that range from 0.1 to 0.9 in interaction weights, here illustrated by *x* > 0 (B), *x* ≥ 0.4 (C), and *x* ≥0.8 (D). The hypothetical matrices are symmetric (*a*_*ij*_ *= a*_*ji*_) and an element of a network does not have a link with itself (diagonal *a*_*ii*_ = 0). Overall, our findings showed that distinct biological systems across scales can have a primary backbone nested structure, but the detection of nested patterns is sensitive to the way we look to the network, i. e., whether considering only the set of strong interactions, or including weak interactions as well. As our findings suggest a relation between nestedness and connectance (S1 Fig), the choice of the link weight threshold used to define an interaction influences the emergence of the pattern: if too permissive, the network is almost fully connected; if too restrictive, the network dismantles into disconnected components as, by reducing the number of interactions, the overlap between nested subsets decrease. Importantly, the interaction strength is not directly related to its biological importance. Weak links are crucial to biological systems, such as occur for infrequent protein [1] and social interactions [2]. Therefore, nestedness is likely to be detected in well-characterized systems whose interactions among the elements are well known and estimated on comprehensive data.
(DOCX)

S1 Table Characterization of the 18 systems encompassing six levels of organization considered in this study and the biological entities or processes depicted by their network representation. (DOCX)
