# Supplementary material for: Nestedness across biological scales
Source: PLoS One. 2017 Feb 6;12(2):e0171691. doi: 10.1371/journal.pone.0171691 (PMC5293200; doi:10.1371/journal.pone.0171691)
Supplement: S1 Fig — (A) UNODF and network connectance (proportion of realized links in relation to possible links); and (B) UNODF and network size (number of nodes). A simple linear regression suggests that UNODF increases with connectance (R2 = 0.73, p<0.0001) but not with size (R2 = 0.07, p = 0.763). Colored points represent empirical nestedness value and whiskers show the 95% confidence interval of UNODF computed for theoretical networks generated using the null model. Values outside of the 95% Confidence Intervals are significant. Note that only nestedness among columns (UNODFc) is displayed, since for all networks (except food webs, Community level) UNODFr = UNODFc. (DOCX) [file pone.0171691.s001.docx]

**Supporting Information:** Cantor et al. Nestedness across biological scales. PLOS ONE.


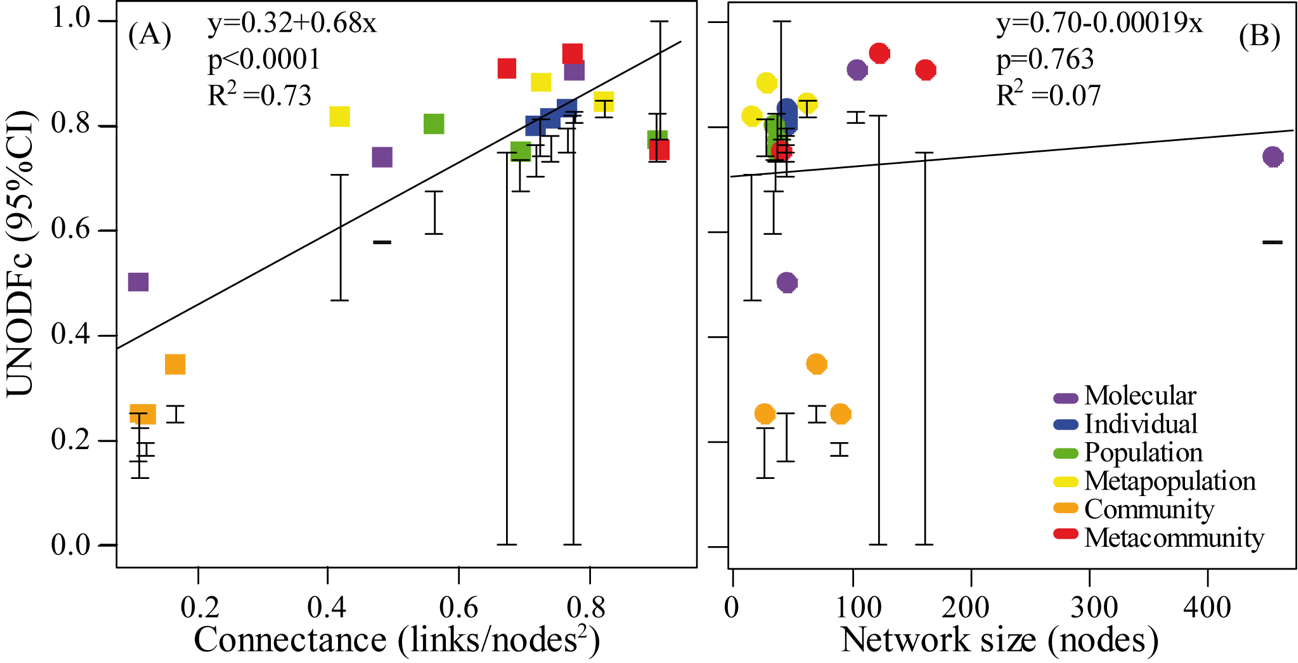


**S1 Fig. Relationship between Unipartite Nestedness (*UNODF*), network connectance and size.** (A) *UNODF* and network connectance (proportion of realized links in relation to possible links); and (B) *UNODF* and network size (number of nodes). A simple linear regression suggests that *UNODF* increases with connectance (*R*^2^=0.73, *p*<0.0001) but not with size (*R*^2^=0.07, *p*=0.763). Colored points represent empirical nestedness value and whiskers show the 95% confidence interval of *UNODF* computed for theoretical networks generated using the null model. Values outside of the 95% Confidence Intervals are significant. Note that only nestedness among columns (*UNODF_c_*) is displayed, since for all networks (except food webs, Community level) *UNODF_r_ = UNODF_c_*.
