# Supplementary material for: Nestedness across biological scales
Source: PLoS One. 2017 Feb 6;12(2):e0171691. doi: 10.1371/journal.pone.0171691 (PMC5293200; doi:10.1371/journal.pone.0171691)
Supplement: S2 Fig — (A) UNODF and size (number of nodes, n) of perfectly nested networks. (B) Adjacency binary matrices (yellow cell = 1, blue = 0) of representative small networks (20 > n > 3) and their respective unipartite nestedness values among columns and rows (UNODFr = UNODFc). The UNODF metric is sensitive to very small networks, but becomes asymptotic for networks with more than 10 nodes. Note that UNODF tends towards—but does not reach—1 because we considered undirected one-mode networks without nodes that interact with themselves. Therefore, the diagonal of the network adjacency matrix A is zeroed (aii = 0). Few cases in which a node can have a link with itself include cannibalistic events in food webs. However, for the sake of generality across biological systems, these cases were disregarded here. (DOCX) [file pone.0171691.s002.docx]

**Supporting Information:** Cantor et al. Nestedness across biological scales. PLOS ONE.


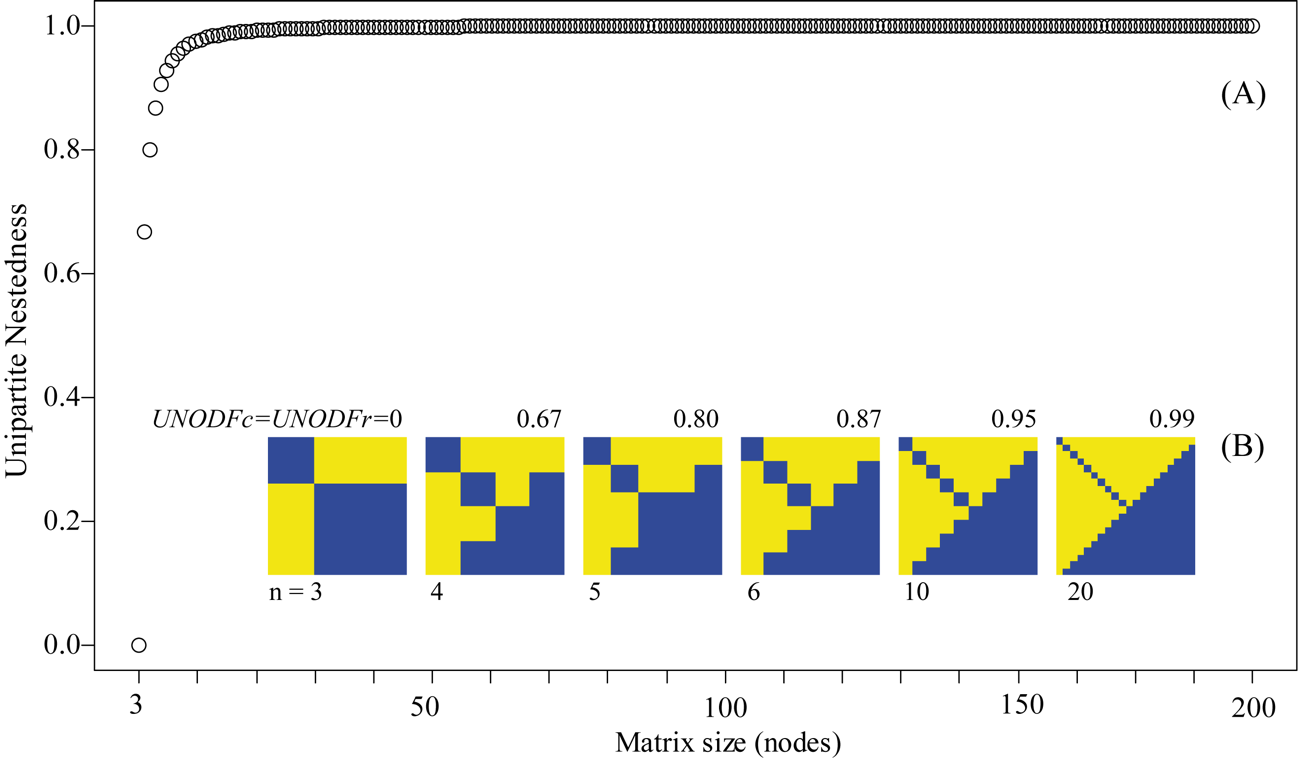


**S2 Fig. Relationship between Unipartite Nestedness (*UNODF*) and size of theoretical nested one-mode networks.** (A) *UNODF* and size (number of nodes, *n*) of perfectly nested networks. (B) Adjacency binary matrices (yellow cell = 1, blue = 0) of representative small networks (20 > *n >* 3) and their respective unipartite nestedness values among columns and rows (*UNODF_r_ = UNODF_c_*). The *UNODF* metric is sensitive to very small networks, but becomes asymptotic for networks with more than 10 nodes. Note that *UNODF* tends towards—but does not reach—1 because we considered undirected one-mode networks without nodes that interact with themselves. Therefore, the diagonal of the network adjacency matrix A is zeroed (*a_ii_*=0). Few cases in which a node can have a link with itself include cannibalistic events in food webs. However, for the sake of generality across biological systems, these cases were disregarded here.
