# Supplementary material for: Nestedness across biological scales
Source: PLoS One. 2017 Feb 6;12(2):e0171691. doi: 10.1371/journal.pone.0171691 (PMC5293200; doi:10.1371/journal.pone.0171691)
Supplement: S3 Fig — Centralization is measured here by 6 metrics (colored lines) for both theoretical (perfectly nested, Barabási, Erdös-Rènyi) and empirical networks (three examples for each of the six biological levels: molecular, individual, population, metapopulation, community, metacommunity; see S1 Table). In (A) the x-axis corresponds to the Unipartite Nestedness metric defined in the main text. In (B), x-axis contains the residuals of the linear regression between Unipartite Nestedness and network connectance (proportion of realized links in relation to possible links) given the positive relationship between them (see Fig 2, S4 Fig). We found no relationship between nestedness and centralization metrics, suggesting that UNODF captures a distinct topological pattern (see also, S4 Fig). Samples are connected simply to make it easier to read the trajectories of each metric. (DOCX) [file pone.0171691.s003.docx]

**Supporting Information:** Cantor et al. Nestedness across biological scales. PLOS ONE.


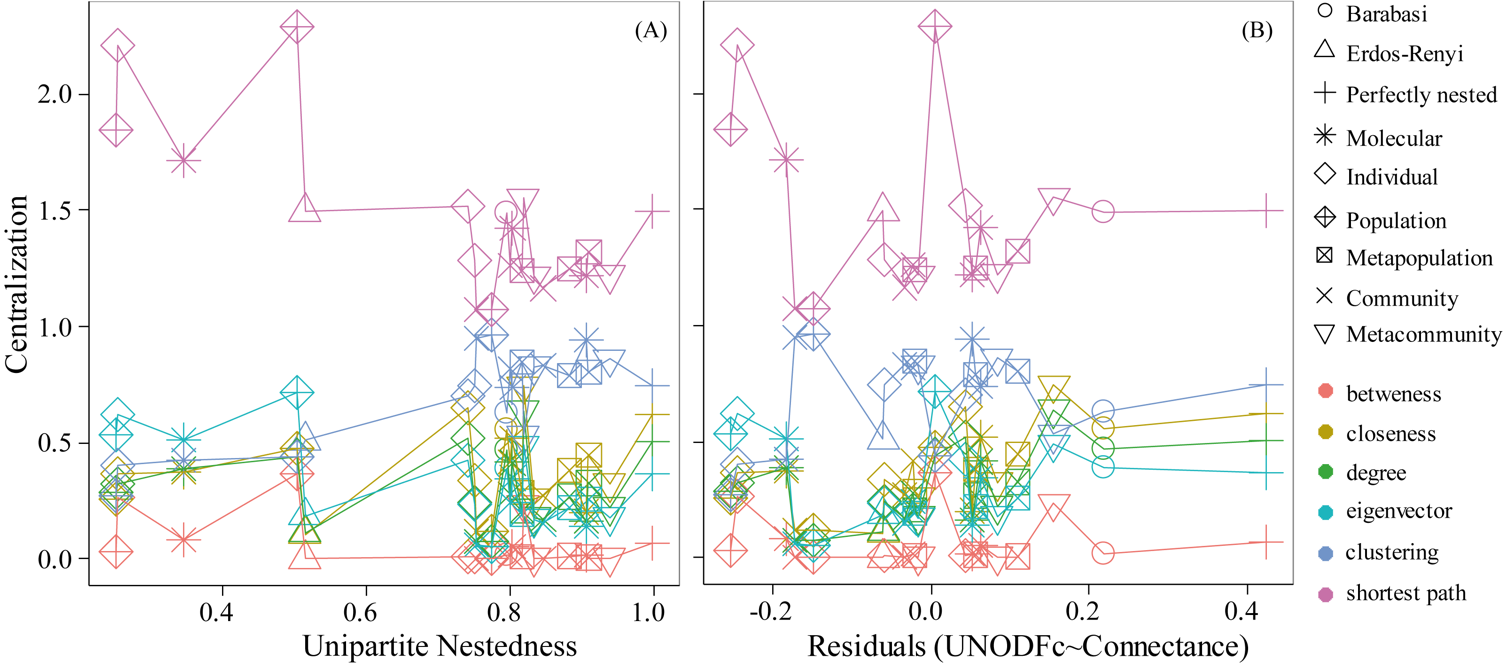


**S3 Fig. Network metrics describing the centralization of the network and Unipartite Nestedness (*UNODF*) in one-mode networks.** Centralization is measured here by 6 metrics (colored lines) for both theoretical (perfectly nested, Barabási, Erdös-Rènyi) and empirical networks (three examples for each of the six biological levels: molecular, individual, population, metapopulation, community, metacommunity; see S1 Table). In (A) the x-axis corresponds to the Unipartite Nestedness metric defined in the main text. In (B), x-axis contains the residuals of the linear regression between Unipartite Nestedness and network connectance (proportion of realized links in relation to possible links) given the positive relationship between them (see Fig 2, S4 Fig). We found no relationship between nestedness and centralization metrics, suggesting that *UNODF* captures a distinct topological pattern (see also, S4 Fig). Samples are connected simply to make it easier to read the trajectories of each metric.
