# Supplementary material for: Nestedness across biological scales
Source: PLoS One. 2017 Feb 6;12(2):e0171691. doi: 10.1371/journal.pone.0171691 (PMC5293200; doi:10.1371/journal.pone.0171691)
Supplement: S4 Fig — Here, we corrected UNODF for network connectance using the residuals of the regression between UNODF and connectance; see S5 Fig. Centralization was described using the first principal component to summarize (A) all 6 centralization metrics, (B) the centrality metrics (degree, betweeness, closeness, eigenvector centrality) and (C) small-world properties (Clustering coefficient and shortest path length). In all cases, the metrics were not related to UNODF (All metrics: R2 = -0.04, p = 0.678; Centrality: R2 = 0.06, p = 0.146; Small world: R2 = 0.01, p = 0.312), suggesting that UNODF captured a topological feature of one-mode network different than centralized networks or with small world properties. (DOCX) [file pone.0171691.s004.docx]

**Supporting Information:** Cantor et al. Nestedness across biological scales. PLOS ONE.


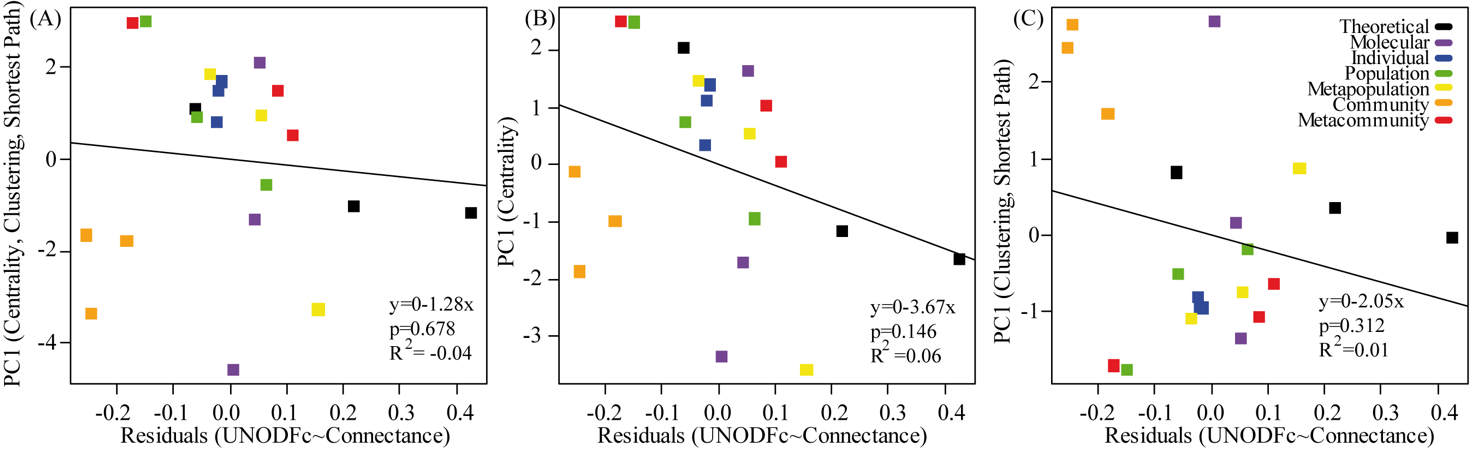


**S4 Fig. Relationship between Unipartite Nestedness (*UNODF*) and network centralization, as given by a simple linear model.** Here, we corrected *UNODF* for network connectance using the residuals of the regression between *UNODF* and connectance; see S5 Fig. Centralization was described using the first principal component to summarize (A) all 6 centralization metrics, (B) the centrality metrics (degree, betweeness, closeness, eigenvector centrality) and (C) small-world properties (Clustering coefficient and shortest path length). In all cases, the metrics were not related to *UNODF* (All metrics: R^2^=-0.04, p=0.678; Centrality: R^2^=0.06, p=0.146; Small world: R^2^=0.01, p=0.312), suggesting that *UNODF* captured a topological feature of one-mode network different than centralized networks or with small world properties.
