# Supplementary material for: Nestedness across biological scales
Source: PLoS One. 2017 Feb 6;12(2):e0171691. doi: 10.1371/journal.pone.0171691 (PMC5293200; doi:10.1371/journal.pone.0171691)
Supplement: S5 Fig — Color code corresponds to the strength of the interaction between elements. Hypothetical square adjacency matrices in which (A) the interactions between elements of a one-mode network are filtered off according to cut-offs (x) that range from 0.1 to 0.9 in interaction weights, here illustrated by x > 0 (B), x ≥ 0.4 (C), and x ≥0.8 (D). The hypothetical matrices are symmetric (aij = aji) and an element of a network does not have a link with itself (diagonal aii = 0). Overall, our findings showed that distinct biological systems across scales can have a primary backbone nested structure, but the detection of nested patterns is sensitive to the way we look to the network, i. e., whether considering only the set of strong interactions, or including weak interactions as well. As our findings suggest a relation between nestedness and connectance (S1 Fig), the choice of the link weight threshold used to define an interaction influences the emergence of the pattern: if too permissive, the network is almost fully connected; if too restrictive, the network dismantles into disconnected components as, by reducing the number of interactions, the overlap between nested subsets decrease. Importantly, the interaction strength is not directly related to its biological importance. Weak links are crucial to biological systems, such as occur for infrequent protein [1] and social interactions [2]. Therefore, nestedness is likely to be detected in well-characterized systems whose interactions among the elements are well known and estimated on comprehensive data. (DOCX) [file pone.0171691.s005.docx]

**Supporting Information:** Cantor et al. Nestedness across biological scales. PLOS ONE.


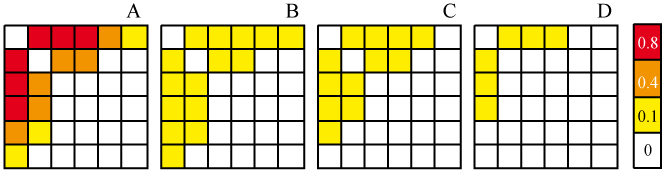


**S5 Fig. Methodological approach to define a binary interaction in weighted networks.** Color code corresponds to the strength of the interaction between elements. Hypothetical square adjacency matrices in which (A) the interactions between elements of a one-mode network are filtered off according to cut-offs (*x*) that range from 0.1 to 0.9 in interaction weights, here illustrated by *x* > 0 (B),$x\geq$ 0.4 (C), and $x\geq$0.8 (D). The hypothetical matrices are symmetric (*a_ij_ = a_ji_*) and an element of a network does not have a link with itself (diagonal *a_ii_* = 0). Overall, our findings showed that distinct biological systems across scales can have a primary backbone nested structure, but the detection of nested patterns is sensitive to the way we look to the network, i. e., whether considering only the set of strong interactions, or including weak interactions as well. As our findings suggest a relation between nestedness and connectance (S1 Fig), the choice of the link weight threshold used to define an interaction influences the emergence of the pattern: if too permissive, the network is almost fully connected; if too restrictive, the network dismantles into disconnected components as, by reducing the number of interactions, the overlap between nested subsets decrease. Importantly, the interaction strength is not directly related to its biological importance. Weak links are crucial to biological systems, such as occur for infrequent protein [1] and social interactions [2]. Therefore, nestedness is likely to be detected in well-characterized systems whose interactions among the elements are well known and estimated on comprehensive data.

[1] Pires MM, Cantor M, Guimarães PR, de Aguiar MA, dos Reis SF, Coltri PP. The network organization of protein interactions in the spliceosome is reproduced by the simple rules of food-web models. Sci Rep. 2015;5:14865. doi: 10.1038/srep14865.

[2] Granovetter MS. The strength of weak ties. Am J Sociol. 1973;1:1360-1380.
