# Supplementary material for: Nestedness across biological scales
Source: PLoS One. 2017 Feb 6;12(2):e0171691. doi: 10.1371/journal.pone.0171691 (PMC5293200; doi:10.1371/journal.pone.0171691)
Supplement: S1 Table — (DOCX) [file pone.0171691.s006.docx]

**Supporting Information:** Cantor et al. Nestedness across biological scales. PLOS ONE.

**S1 Table.** Characterization of the 18 systems encompassing six levels of organization considered in this study and the biological entities or processes depicted by their network representation.

|  | Biological system | Level of organization | Nodes | Interaction | Weighted links | Network size (nodes) | Connectance (realized/  possible links) | Main References |
| --- | --- | --- | --- | --- | --- | --- | --- | --- |
| 1 | Yeast spliceosome proteins | Molecular | proteins | physical association | Experimental evidence for interaction | 103 | 0.78 | [1] |
| 2 | *Caenorhabdtis elegans* genes | Molecular | target genes | shared interactions with query genes | Simpson index | 454 | 0.48 | [2,3] |
| 3 | Yeast nuclear exosome proteins | Molecular | proteins | physical association | Experimental evidence for interaction | 44 | 0.10 | [4,5] |
| 4 | Cranium morphology of human males | Individual | anatomical landmarks (morphometric variables) | correlations between cranial measurements | Pearson's correlation | 44 | 0.73 | [6] |
| 5 | Cranium morphology of human females | Individual | anatomical landmarks (morphometric variables) | correlations between cranial measurements | Pearson's correlation | 44 | 0.71 | [6] |
| 6 | Cranium morphology of human males in Europe | Individual | anatomical landmarks (morphometric variables) | correlations between cranial measurements | Pearson's correlation | 44 | 0.76 | [6] |
| 7 | Guiana dolphin society | Population | individuals | social relationship | Half-weight association index | 33 | 0.56 | [7] |
| 8 | Bottlenose dolphin society | Population | individuals | social relationship | Half-weight association index | 35 | 0.69 | [8] |
| 9 | Spotted hyena social clan | Population | individuals | social relationship | Half-weight association index | 35 | 0.62 | [9-11] |
| 10 | Insular frog populations | Meta population | population | Genetic similarity | 1-R_ST_ total allelic variance | 27 | 0.72 | [12,13] |
| 11 | Global human populations | Meta population | population | Genetic similarity | 1-R_ST_ total allelic variance | 62 | 0.82 | [14,15] |
| 12 | Insular sparrow populations | Meta population | population | Genetic similarity | 1-R_ST_ total allelic variance | 15 | 0.41 | [16,17] |
| 13 | Food web of the Mangrove estuary in wet season | Community | species | Trophic | Energy transfer | 70 | 0.16 | [18] |
| 14 | Food web of the Narragansett Bay estuary | Community | species | Trophic | Energy transfer | 26 | 0.11 | [19] |
| 15 | Food web of the Florida Bay in dry season. | Community | species | Trophic | Energy transfer | 89 | 0.12 | [18] |
| 16 | Reef fishes communities in a biogeographical region | Meta community | genera | Co-occurrence in sampled sites | Bray-Curtis similarity | 158 | 0.18 | [20] |
| 17 | Reef fishes communities in a biogeographical region | Meta community | functional group (length+ diet) | Co-occurrence in sampled sites | Bray-Curtis similarity | 39 | 0.46 | [20] |
| 18 | Reef fishes communities in a biogeographical region | Meta community | functional group (length+ diet+ mobility+ school size) | Co-occurrence in sampled sites | Bray-Curtis similarity | 120 | 0.24 | [20] |

**Supplementary References**

[1] Szklarczyk D, Franceschini A, Kuhn M, Simonovic M, Roth A, Minguez P, et al. The STRING database in 2011: functional interaction networks of proteins, globally integrated and scored. Nucleic Acids Res. 2011;39:D561-D568. doi: 10.1093/nar/gkq973

[2] Byrne AB, Weirauch MT, Wong V, Koeva M, Dixon SJ, Stuart JM, Roy PJ. A global analysis of genetic interactions in *Caenorhabditis elegans*. J Biol. 2007;6:8. doi: jbiol.com/content/6/3/8

[3] Chatr-aryamontri A, Breitkreutz BJ, Oughtred R, Boucher L, Heinicke S, Chen D, et al. The BioGRID interaction database: 2015 update. Nuc Acids Res. 2014;43:D470-D478. doi: 10.1093/nar/gku1204

[4] Krogan NJ, Cagney G, Yu H, Zhong G, Guo X, Ignatchenko A, et al. Global landscape of protein complexes in the yeast *Saccharomyces cerevisiae*. Nature. 2006;440:637-643. doi:10.1038/nature04670

[5] Orchard S, Kerrien S, Abbani S, Aranda B, Bhate J, Bidwell S, et al. Protein interaction data curation: the International Molecular Exchange (IMEx) consortium. Nature Meth. 2012;9:345-350. doi:10.1038/nmeth.1931

[6] Howells WW. Cranial variation in man: A study by multivariate analysis of patterns of difference among recent human populations. Papers Peabody Museum Archaeol Ethnol. 1973;67:1-259.

[7] Cantor M, Wedekin LL, Guimarães PR Jr, Rossi-Santos MR, Simões-Lopes PC. Disentangling social networks from spatiotemporal dynamics: the temporal structure of a dolphin society. An Behav. 2012;84:641-651. doi: 10.1016/j.anbehav.2012.06.019

[8] Daura-Jorge FG, Cantor M, Ingram S, Lusseau D, Simões-Lopes PC. The structure of a bottlenose dolphin society is coupled to a unique foraging cooperation with artisanal fishermen. Biol Lett. 2012;8:702-705. doi:10.1098/rsbl.2012.0174

[9] Smith JE, Kolowski JM, Graham KE, Dawes SE, Holekamp KE. Social and ecological determinants of fission-fusion dynamics in the spotted hyaena. An Behav. 2008;76:619–636. doi:10.1016/j.anbehav.2008.05.001

[10] Holekamp KE, Smith JE, Strelioff CC, Van Horn RC, Watts HE. Society, demography and genetic structure in the spotted hyena; 2011. Database: Dryad Digital Repository [Internet]. Accessed: http://dx.doi.org/10.5061/dryad.tg582

[11] Holekamp KE, Smith JE, Strelioff CC, Van Horn RC, Watts HE. Society, demography and genetic structure in the spotted hyena. Mol Ecol. 2012;21:613-632. doi: 10.1111/j.1365-294X.2011.05240.x

[12] Wang S, Zhu W, Gao X, Li X, Shaofei Y, Liu X, Yang J, Gao Z, Li Y. Population size and time since island isolation determine genetic diversity loss in insular frog populations; 2013. Database: Dryad Digital Repository [Internet]. Accessed: http://dx.doi.org/10.5061/dryad.dq4g5

[13] Wang S, Zhu W, Gao X, Li X, Shaofei Y, Liu X, Yang J, Gao Z, Li Y. Population size and time since island isolation determine genetic diversity loss in insular frog populations. Mol Ecol. 2014;23:637-648. http://dx.doi.org/10.1111/mec.12634

[14] Wang S, Lewis Jr CM, Jakobsson M, Ramachandran S, Ray N, Bedoya G, et al. Genetic variation and population structure in Native Americans. PLoS Genet. 2007;3:e185. doi: 10.1371/journal.pgen.0030185

[15] Rosenberg NA, Mahajan S, Ramachandran S, Zhao C, Pritchard JK, Feldman MW. Clines, clusters, and the effect of study design on the inference of human population structure. PLoS Genet. 2005;1:e70. doi:10.1371/journal.pgen.0010070

[16] Baalsrud HT, Sæther B, Hagen IJ, Myhre AM, Ringsby TH, Pärn H, Jensen H. Effects of population characteristics and structure on estimates of effective population size in a house sparrow metapopulation; 2014. Database: Dryad Digital Repository [Internet]. Accessed: http://dx.doi.org/10.5061/dryad.nb260

[17] Baalsrud HT, Sæther BE, Hagen IJ, Myhre AM, Ringsby TH, Pärn H, Jensen H. Effects of population characteristics and structure on estimates of effective population size in a house sparrow metapopulation. Mol Ecol. 2014;23:2653-2668. doi: 10.1111/mec.12770

[18] Christian R, Luczkovich J. Organizing and understanding a winter’s seagrass foodweb network through effective trophic levels. Ecol Model. 1999;117:99-124. doi:10.1016/S0304-3800(99)00022-8

[19] Monaco ME, Ulanowicz RE. Comparative ecosystem trophic structure of three U.S. mid-Atlantic estuaries. Mar Ecol Prog Ser. 1997;161:239-254.

[20] Floeter SR, Krohling W, Gasparini JL, Ferreira CE, Zalmon IR. Reef fish community structure on coastal islands of the southeastern Brazil: the influence of exposure and benthic cover. Environ Biol Fishes. 2007;78:147-160. doi: 10.1007/s10641-006-9084-6
